# Low intensity technology-delivered cognitive behavioral therapy for obsessive-compulsive disorder: a meta-analysis

**DOI:** 10.1186/s12888-021-03272-5

**Published:** 2021-06-30

**Authors:** Laura Marie Hoppen, Nora Kuck, Paul-Christian Bürkner, Eyal Karin, Bethany M. Wootton, Ulrike Buhlmann

**Affiliations:** 1 University of Münster, Fliednerstr. 21, 48149 Münster, Germany; 2 Cluster of Excellence SimTech, University of Stuttgart, Stuttgart, Germany; 3 Macquarie University, Sydney, Australia; 4 University of Technology Sydney, Sydney, Australia

**Keywords:** Obsessive-compulsive disorder, OCD, Technology-delivered cognitive behavioral therapy, CBT, Meta-analysis

## Abstract

**Background:**

Cognitive behavioral therapy (CBT) is a well-established treatment for people suffering from obsessive-compulsive disorder (OCD) and technology-based CBT applications are an emerging treatment option for people with OCD. These applications involve treatment protocols with automated content delivery and relatively low clinical contact. Whilst such CBT applications are promising, however, further investigation is needed to establish the efficacy of this treatment approach for individuals with OCD. The aim of the present study was to review the efficacy of technology-delivered CBT with minimal clinician support for OCD using a meta-analytic approach.

**Methods:**

Randomized controlled trials (RCT) were identified through PsycINFO, Medline and Scopus resulting in 18 eligible studies (*n* = 1707). Control conditions comprised both passive (namely no treatment, other treatments and waitlist controls) and active. Measurement of OCD symptoms improvement was the outcome in each study.

**Results:**

Participants in the technology-delivered CBT group scored lower on Yale-Brown Obsessive-Compulsive Scale (Y-BOCS) (*g* = − 0.59, 95% CI = [− 0.99, − 0.18], *p* = 0.01), Y-BOCS and Dimensional Obsessive-Compulsive Scale (DOCS) combined (*g* = − 0.55, 95% CI = [− 0.87, − 0.24], *p* = 0.003) and Obsessive-Compulsive-Inventory-Revised (OCI-R) (*g* = − 0.36, 95% CI = [− 0.62, − 0.09], *p* = 0.02) at post-treatment than passive control groups. There were no significant findings when compared to controls with other treatments.

**Conclusions:**

This meta-analysis suggests that technology-delivered CBT with low personal contact intensity, relative to passive control groups, is an efficacious and promising treatment option for individuals with OCD. Further research is needed to allow for a comparison with control groups with other treatments.

**Supplementary Information:**

The online version contains supplementary material available at 10.1186/s12888-021-03272-5.

## Background

### Obsessive-compulsive disorder (OCD)

Obsessive-compulsive disorder (OCD) is a common mental disorder with a lifetime prevalence of 2–3% [1, 2]. The disorder is characterized by obsessive, unwanted and recurrent thoughts (obsessions) and repetitive and time-consuming behaviors (compulsions) [3]. The disorder results in a considerable reduction quality of life [4] and is associated with significantcosts for the individual and society [5].

### Types of treatment for OCD

Randomized controlled trials (RCTs) have demonstrated the efficacy of different types of treatments for OCD including exposure and response prevention (ERP), cognitive therapy [6], and pharmacotherapy with Selective Serotonin Reuptake Inhibitors (SSRIs) [7]. Cognitive-behavioral therapy (CBT) including ERP is an evidence-based therapeutic intervention for OCD [8] and is recommended by expert guidelines (National Institute for Health and Care Excellence; NICE, 2005). Rosa-Alcázar, Sánchez-Meca, Gómez-Conesa, and Marín-Martínez (2008) demonstrated that both cognitive restructuring (CR) and ERP alone, and in combination, are effective in the treatment of OCD [9].

### Types of treatment delivery

Alongside the availability of multiple treatment models, the application of treatment can also differ in several aspects. Treatment for OCD is generally delivered by a therapist in face-to-face sessions, however more recently, bibliotherapy-, and technology-delivered interventions [i.e., computerized CBT (cCBT) or internet-based CBT (iCBT)] have also been used. There are mixed results. A recent meta-analysis demonstrated that face-to-face CBT, iCBT and bCBT showed no evidence for differences in benefits, however, face-to-face treatment necessitated more clinician time than the other types of delivery [10]. A more recent review questioned the non-inferiority of technology-delivered CBT to face-to-face delivery for anxiety disorders because of restricted evidence from RCTs [11].

Technology-delivered interventions have the potential to overcome structural and personal barriers to treatment, or they can be a supplement in the context of a face-to-face weekly intervention with a therapist. Due to the cost-advantages of technology-delivered interventions [12], as well as the difficulty in accessing well-trained therapists in many countries (e.g., Germany [13], England and Wales [14]), technology-delivered CBT is an important addition to the treatment of mental health disorders. Importantly, even though patients may prefer face-to-face sessions instead of internet-based treatments [15], technology-delivered CBT results in a high rate of satisfaction from consumers [10].

Many different types of technology can be used to deliver CBT for OCD. For instance, interventions can be delivered through a computer, which may or may not be linked to the internet, personal digital assistants, virtual reality devices, an interactive voice response (IVR) system, a PDF file sent via email, web-cameras, CD-ROMS, DVDs, telephones, and mobile phone software applications [16]. Technology-delivered CBT can also be provided as either a clinician-guided or unguided intervention. Newman, Szkodny, Llera, and Przeworski (2011) suggest four different levels of therapist contact including: (1) high contact intensity (HCI) (a defined number of contacts with a clinician, remote intervention provides an augmentation of the therapist sessions); (2) reduced contact intensity (RCI) (less assistance than usual therapy, providing an assistance in terms of the application of a certain therapeutic techniques while the assistance involves more than 1.5 h of a therapist’s time); (3) self-help with assistance to ensure engagement with the tool (ASH) (no therapeutic input regarding therapeutic techniques, only contact for reminding to check-in, explaining the tool and/or the basic principle while in total the assistance does not involve more than 1.5 h); and, [4] pure self-help (PSH) (only contact with a clinician for the assessment, otherwise no support at all during fully automated intervention programs) [17].

### Evidence about technology-delivered CBT for OCD

Several meta-analyses have recently been conducted exploring the efficacy of technology-delivered CBT for OCD [18–21]. Pozza etal. (2014) published a meta-analysis that includeda variety of different study designs (i.e., pre−/ post-test, one-group designs, and RCTs) [18]. Regarding sample characteristics, participants needed to have a primary OCD diagnosis, however, there was no restriction of participants’ age, thus trials with children and adolescents were also included. The main focus of the meta-analysis by Pozza et al. (2014) was CBT delivered through computers, excluding telephone- or web-camera-delivered interventions [18].

Dèttore et al. (2015) conducted a meta-analysis integrating samples with a primary OCD diagnosis (both adults and children/adolescents) [19]. In this meta-analysis, Dètorre and colleagues (2015) examined pure self-help CBT, CBT with reduced therapist contact (e.g., only via e-mail) and telephone or web-camera-delivered CBT [19]. Overall, eight RCTs were included in the final analysis. Findings of both meta-analyses, Pozza et al. (2014) and Dètorre et al. (2015), indicated a large effect favoring remote CBT over control conditions (*d* = 0.82, *p* = 0.001) [18, 19]. For both meta-analyses, the number of included studies was very small (*N* = 8). Thus, findings should be considered with caution.

Pearcy et al. (2016) analyzed all trials with available technology-delivered self-help interventions for OCD, and thus included interventions that were not cognitive-behavioral in orientation [20]. Additionally, the study examined if treatment outcomes were influenced by the amount of therapeutic contact provided. Results indicated that an increase of therapeutic contacts was associated with an increase of effect sizes. Given this meta-analysis included non-CBT interventions it is difficult to ascertain the effect of CBT oriented technology delivered interventions for OCD.

Wootton (2016) performed the only meta-analysis that quantified outcomes of remote treatments for OCD by comparing types of remote treatments and the contact intensity during treatments [21]. The author described videoconferencing-administered CBT (vCBT) and telephone-administered CBT (tCBT) as high intensity remote treatments and cCBT as well as iCBT and bibliotherapy administered CBT (bCBT) as low intensity remote treatments. Moreover, she compared guided and self-guided treatments. Thus, the main strength of this meta-analysis is that remote treatments were split into their characteristics. The results indicated that remote treatment seems to be as efficacious as face-to-face treatment. However, a limitation of this meta-analysis is that open trials were included as well and findings might be outdated now.

Overall, the current state of research indicates reduced efficacy and effectiveness of technology-delivered CBT when provided without a regular clinician support (e.g., [22, 23]). However, pure self-help interventions result in significant reduction of symptoms as well [24, 25]. As different intensities of personal contact during a remote treatment are increasingly becoming available, questions remain how much guidance is needed to maintain an efficacy of treatment for OCD patients. Moreover, after considering the most recent published meta-analyses, it becomes clear that there is a need to investigate the efficacy of technology-delivered treatments for OCD using controlled studies.

### Rationale of the current meta-analysis

The aim of the current paper is to extend earlier meta-analyses and investigate (a) whether low intensity technology-delivered CBT results in significant improvement in OCD symptoms from pre- to post-treatment compared to control conditions; (b) the efficacy of low intensity technology-delivered interventions at post-treatment compared to either passive, namely wait-list, or active controls on OCD symptoms; (c) whether defined study characteristics moderate the symptom reduction. Additionally, we were interested if type of technology, contact with a therapist/ clinician or how OCD diagnosis was assessed influenced the efficacy of low intensity technology-delivered interventions or rather symptom reduction. We defined the main research question describing the Population, Intervention, Comparison, Outcome, and Study design (PICOS) in accordance with the recommendations by the Preferred Reporting Items for Systematic Reviews and Meta-analyses (PRISMA) group [26]. The question was “In patients with OCD (P), does technology-delivered low-intensity CBT (I), compared to passive (no treatment or waitlist) and active control conditions (C), improve OCD symptom severity at post-assessment and from pre- to post-assessment (O) in randomized controlled trials (S)?”

To the best of our knowledge, this is the first meta-analysis to investigate the efficacy of low intensity technology-delivered interventions for OCD using only RCTs. One should also highlight that this study is un up-to-date synthesis as we included recently (*N* = 8) published studies.

## Methods

All the materials including screening sheet, data extractions sheet, study quality assessments and analyses are available from our Open Science Framework (OSF) data repository (https://osf.io/7upt4/?view_only=34f63ec15df545f0a8abbc6bdf14d77d).

### Eligibility criteria

PRISMA statement criteria [26] were the basis for reporting the present meta-analysis. There is no review protocol online. The inclusion and exclusion criteria were established prior to the study selection process and are described as follows:

#### Types of participants

In the study samples, participants needed to be adults (mean age > 18 years) and to have OCD symptoms according to a standardized classification system [3], prior diagnosed by a mental health specialist (e.g., [27, 28]), self-reported OCD symptoms (i.e., [29, 30]) or a score measuring OCD symptoms at least on a mild level (i.e., a Y-BOCS score over 7). There were no restrictions in terms of medication use.

#### Types of interventions

Studies were included if they conducted trials with technology-delivered cognitive behavioral therapy such as cCBT, iCBT or bCBT. The latter could be a PDF file sent or provided via a technology (e.g., computer or mobile phone). Interventions were defined as CBT if they included typical CBT elements such as cognitive components (e.g., cognitive restructuring), ERP or components of the third wave of CBT. Moreover, studies needed to provide interventions with low personal contact intensity. According to Newman et al. (2011) our definition of a low contact intensity included category [3] self-help with assistance to ensure engagement with the tool (ASH) and [4] pure self-help (PSH) [17]. According to proposed guidelines by Newman et al. (2011) and Andersson, Calbring, Berger, Almlöv, and Cuijpers (2009) [17, 31], we defined a maximal timeframe of 100 min for category [3] in terms of phone calls. In other words, time spent for therapists’ responses without time latencies (synchronous contact) is not more than 100 min per participant and per intervention independent of the intervention length. Additionally, contacts involved no face-to-face sessions or video calls. No time restrictions were made with regard to responses with time latencies (asynchronous contact) such as written answers or recorded answers (e.g., IVR systems). Asynchronous contacts did not provide crisis interventions or prompt responses and resulted in less personal and situational influences by a therapist as the answers were recorded. Group conditions which provide more than 100 min in total or/and included face-to-face sessions or video calls were defined as an active control group condition.

As explained above, our definition for a low contact intensity included self-help with assistance to ensure engagement with the tool (ASH) and pure self-help (PSH) with a maximal timeframe of 100 min per participant and per intervention independent of the intervention length.

#### Treatment models

Comparison was drawn between cCBT, iCBTor bCBTand an active or passive control condition. Control conditions with other treatments were face-to-face CBT, video-based CBT, phone-based CBT with phone calls over 100 min during the treatment, only CBT psychoeducation, relaxation or attention treatments or other interventions than CBT. Passive control conditions were considered as wait-list groups or no treatment conditions.

#### Main outcomes

Included studies needed to report outcomes on measures of OCD symptoms (e.g., Yale-Brown Obsessive-Compulsive Scale (Y-BOCS) [32]; Obsessive-Compulsive-Inventory-Revised (OCI-R) [33]; Dimensional Obsessive-Compulsive Scale (DOCS) [34]).The outcomes of interest were standardized mean differences of symptom severity. To calculate effect sizes, studies had to provide pre- and post-treatment means and standard deviations. When these values and information were not provided, authors were contacted.

#### Design and backgrounds

Studies needed to be published in either German or English. Only studies with randomized controlled trials were included. Thus, open trials, preliminary studies, case reports or trials based on the same data were excluded. Furthermore, studies were required to investigate the efficacy of an intervention. Hence, effectiveness studies were excluded. By definition, studies conducting interventions under real world day-to-day circumstancesare called effectiveness studies. Conducting interventions under ideal, high controlled conditionsis referred to as efficacy studies [35]. To adequately compare trials, we focused solely on efficacy trials. However, until now the research status does not provide many effectiveness studies. Additionally, the question arises if we can appropriately distinguish between effectiveness and efficacy studies since internet interventions can be conducted in clinical settings and in an extensive public health setting [36]. However, in order to attempt the distinction between effectiveness and efficacy studies, we focused during the full-text screening on references to effectiveness studies or otherwise called pragmatic trials.

### Search procedure

Up to March 2021 electronic databases PsycINFO, Medline, and Scopus were searched for eligible studies. For identifying additional studies search was made through reference lists of previous published meta-analyses and reviews [18–21, 37] and through contacting corresponding authors to request any further unpublished studies.

The following search term was used for Medline and PsycINFO: obsessive compulsive or OCD AND internet-based or internet-delivered or online or technology-delivered or computer-based or remote or app or mobile AND therapy or treatment or intervention or psychotherapy or CBT. For the database Scopus there were used the following search terms: OCD AND online* OR technology* OR internet* OR computer* AND CBT OR intervention* OR treatment*.

#### Study selection and data extraction process

Study selection and the development of the Excel data sheet was conducted by the first author (LNH). First, title and abstract screening resulted in removing duplications and unsuitable studies according to inclusion criteria. Second, full texts were identified in terms of inclusion criteria. When necessary study information and/ or statistics were not available, authors have been contacted for providing missing data. The following data was extracted from each study: (a) characteristics of the sample (sample size, age, percentage of females, diagnosis instrument, country), (b) inclusion and exclusion criteria, (c) type of controls (active, passive, contact intensity with therapists), (d) type of interventions (technology delivery, length, contact intensity with therapists in minutes), (e) outcome measures, (f) number of completers and dropouts, (g) estimation methods (ITT, completer analyses), (h) results (means, standard deviations, sample sizes, effect sizes, F values, t values, *p* values, including assessment times such as pre and post). Completers were identified as participants who completed assessments after the treatment (post-assessment). The first two authors (LNH and NK) independently extracted the data from included studies. If there was any disagreement, it was discussed and resolved by consensus in meetings. The coding agreement of the relevant variables (M, SD, N) was 98%.

### Study quality

Study quality was assessed focussing on criteria for judging risk of bias developed by the Cochrane Collaboration [38]. Study quality was assessed by independent evaluators and discrepancies were solved until full agreement was obtained. Assessed criteria were the following: selection bias (allocation concealment and random sequence generation), attrition bias (incomplete outcome data focussing on OCD symptoms outcome), detection bias (blinding of OCD symptoms assessment), and reporting bias (selective reporting). Blinding of participants and personnel was not included in the study quality assessment as this criterion is hardly possible to fulfil in psychotherapy research. In line with Bennet et al. (2019) [39], detection bias regarding OCD self-report measures were considered to be based on a low risk of bias. In particular, this conclusion is made as self-administered versus clinician-administered versions of the Y-BOCS show identical capability to differentiate OCD patients from other subjects [40, 41]. The response *unclear* was used when no further information was provided or when descriptions did not provide enough information. Quality criteria were rated either *high* (coded with 2), *low* (coded with 0) or *unclear* (coded with 1). For an examination of the relationship between risk of bias and effect sizes, final risk of bias scores for each study were entered as an additional moderator. Moreover, we conducted a sub analysis including only studies with low risk of bias.

### Data analysis

#### Summary measures

All analyses were conducted in the programming language R (version 3.6.1; R Core Team, 2019) and specifically with the help of the R package metafor [42]. For all statistical tests, we set a 5% significance level. When directed hypotheses were obtainable, tests were one-tailed.

To compute effects sizes, we used the following procedure. The standard deviations of the post – pre differences per group were computed using basic mathematical rules of (co) variances and assuming a correlation of *r* = 0.5 between pre and post scores (see online supplement for the corresponding code). Correlations around *r* = 0.5 are commonly found in intervention studies and have been used in other meta-analyses before (e.g., [43]). In a sensitivity analysis, we set the correlation to *r* = 0, which leads to the largest standard deviations and thus to the most conservative estimates as the uncertainty in the effect sizes is very likely overestimated (results were numerically slightly different but qualitatively equivalent; see online supplement). Based on the obtained post-pre mean differences and corresponding standard deviations per group, Hedges’ g was computed between experimental and control group. Hedges’ g corrects for the small positive bias in Cohen’s d. For both effect sizes, the following classification are typically used: small effect (*d* = 0.2), medium (*d* = 0.5) and large (*d* = 0.8) effects [44]. Intention to treat (ITT) data was used when available, however, completer data was used when ITT was not available.

Regarding OCD instruments, there is a correlation of 0.92 between YBOCS and DOCS scores, providing strong evidence of convergent validity for assessing OCD instruments (r = 0.92 [45]). Thus, if studies reported both DOCS and YBOCS measures, a mean effect estimate of both OCD instruments was calculated. The correlation between OCI-R and Y-BOCS indicates a medium degree (*rs* = .43 [46]). Abramowitz and colleagues (2010) supposed that the lower degree between OCI-R and Y-BOCS might be due to the measurement of distress associated with specific OC symptoms, whereas the Y-BOCS measures the global symptom severity [34]. Therefore, OCI-R was analysed separately. Comparison between the self-report rating scale of YBOCS versus YBOCS interview report a strong agreement [47] thus both types were analyzed together.

Throughout, we were applying random effects meta-analytic models as studies could a-priori be assumed to differ systematically in their true effect sizes because of differences in, for example, study design or investigated samples [48]. This assumption was later confirmed in the obtained results as we found significant heterogeneity between effect sizes. Heterogeneity between studies occurs as studies vary regarding experimental design, outcome assessment, and treatment characteristics [35]. Statistical heterogeneity results when the true effects of the varying studies lead to a higher discrepancy than expected coincidentally. We applied the statistical heterogeneity by using I^2^ method by Higgins and Thompson (2002) and also investigated the between study and between effect size variance estimates directly [35]. Forest plots were used visualize results and detect potential outliers.

#### Moderator analysis

Moderator analyses using meta-regression [30] for comparison between remote treatments versus passive controls were performed with study characteristics of underlying theoretical interest. The following moderators were coded: contact with a therapist/ clinician, OCD diagnosis assessed by a measurement based on DSM, meanage, gender, type of technology, duration of intervention and study quality. For the variables support condition and OCD diagnosed according to a DSM version with a clinical structured interview, we coded dummy variables.

#### Publication bias

Research articles with significant results are likelier published than with insignificant or otherwise less convenient results [49]. Thus, publication bias can lead to an overestimation of effects. We tried to minimize potential for publication bias as much as possible by actively searching for unpublished results, in particular dissertations. However, dissertation papers (with unpublished data) were not included as we could not find eligible dissertations. To investigate the likelihood and the extent of a publication bias (or other sources of skewness in the effect size distribution), we generated Funnel plots and applied Egger’s regression tests [50].

## Results

### Study selection

**Fig. 1:**
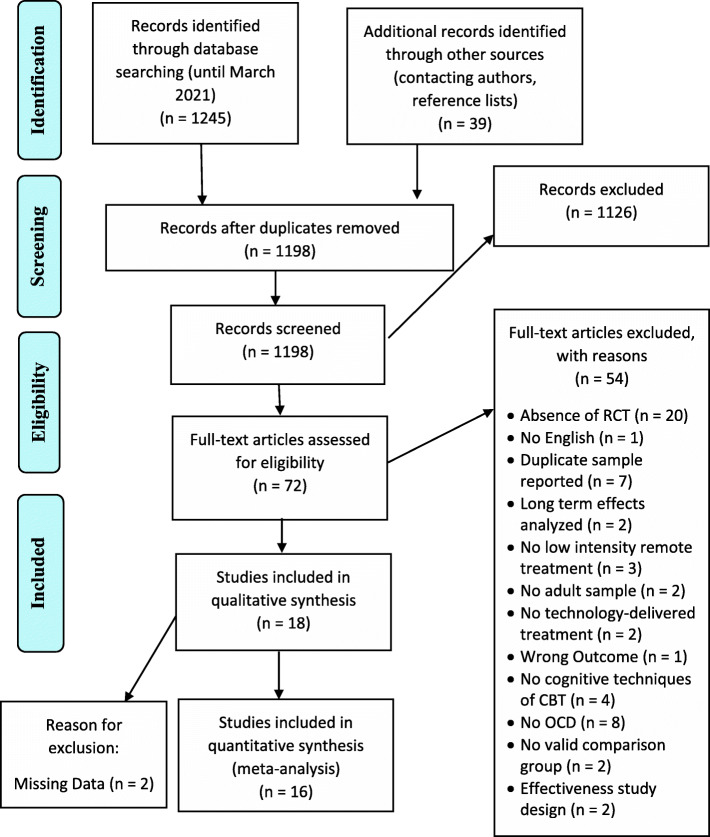
PRISMA study flow chart

A total of 1284 records were identified through the electronic and the search through additional sources. After removing duplicates, 1198 records remained. Of these 1198, 1126 were excluded after a thorough title and abstract screen as these publications either utilized another study design than RCT or presented with irrelevant topics. For the remaining 72 studies, a full-text screen was performed. Of these, 18 studies met inclusion criteria and were included in the present analysis. The selection process is presented in the PRISMA flow chart (Fig. 1).

### Sample and intervention characteristics

An overview of the 18 included studies is provided in Table 1. Included RCTs comprised only adult samples from the following countries: Australia (*k* = 4), Russia (*k* = 2), Germany (*k* = 5), Sweden (*k* = 1), USA (*k* = 1), Norway (*k* = 1), Italy (*k* = 1), and participants from different Arabic speaking countries (*k* = 1). Publication year ranged from 2002 to 2021, and all studies were published in English. A total of 1707 subjects (thereof a total of 1546 subjects for the quantitative synthesis) were included in the studies and underwent randomization, whereas a total of 1171 (thereof a total of 1073 subjects for the quantitative synthesis) completed the post-test-assessment (low contact intensity technology-delivered interventions: *N* = 522 (thereof a total of 475 subjects for the quantitative synthesis); control conditions *N* = 682 (thereof a total of 630 subjects for the quantitative synthesis)). The mean age across all 18 studies was 34,45 years and the mean percentage of female participants was 63,91% (i.e., unweighted means).

**Table 1:** Characteristics of included studies

Study	Origin of sample	N	%F	Mean Age (years)	OCD according to	OCD symptoms outcome	Type of t.-d. treatment	Duration of treat-ment (weeks)	Control group condition	Guidance provided by the therapist (type, total minutes)
**Andersson et al., 2012** [51]	Sweden	101	66.3	34	DSM-IV-TR	Y-BOCS, OCI-R self-reported	iCBT	10	Other treatments (online non-directive supportive therapy)	Contact if needed via messages, 129
**Greist et al., 2002** [52]	USA	176	42	39	DSM-IV	Y-BOCS-SR	BT Steps	10	Other treatments (clinician guided face-to-face), active (relaxation daily, guided by a manual and an audiotape)	Recorded answer to a personal message within 72 h and recorded voice files (IVR-system), 140.5
**Hauschildt, Schröder & Moritz, 2016** [53]	Germany	128	67	39	DSM-IV	Y-BOCS	myMCT	4	Other treatments (psychoeducation)	No support, 0
**Herbst et al., 2014** [54]	Germany	34	64.7	35.7	DSM-IV	Y-BOCS-SR, OCI-R	iCBT	8	Passive (waitlist)	Feedback via secure web-based communication system, 462
**Kyrios et al., 2018** [55]	Australia	179	65.7	33.4	DSM-IV-TR	Y-BOCS	iCBT	12	Other treatments (iPMR)	Email, max. 180
**Moritz et al., 2016** [56]	Russia	89	48.3	25.2	Mental health specialist	Y-BOCS-SR, OCI-R	myMCT	6	Passive (waitlist)	No support, 0
**Wootton et al., 2013** [57]	Australia	36	93.3	39	DSM-IV	Y-BOCS, DOCS	iCBT	8	Passive (waitlist)	Twice weekly therapist contact, 88.63
**Schneider et al., 2014** [58]	Germany	65	55.8	37.3	Mental health specialist	Y-BOCS-SR, OCI-R	COMET	4	Passive (waitlist)	No support, 0
**Moritz et al., 2010** [28]	Germany	86	72.1	34.5	Mental health specialist	Y-BOCS-SR, OCI-R	myMCT	4	Passive (waitlist)	Only answers if questions, 0
**Moritz et al., 2018** [27]	Germany	70	71.4	38.8	Mental health specialist	Y-BOCS-SR, OCI-R	myMCT	6	Passive (waitlist)	No support, 0
**Moritz & Jelinek, 2011** [59]	Germany	46	56.5	36.2	Mental health specialist	Y-BOCS-SR, OCI-R	Associa-tion Splitting	4	Passive (waitlist)	Only answers if questions, 0
**Mahoney et al., 2014** [60]	Australia	86	60	39	DSM-IV	DOCS	iCBT	10	Other treatments (TAU which is no CBT treatment, e.g. 13 /35 medication treatment)	Scheduled personalised emails, 6
**Moritz et al., 2019** [29, 30]	Different Arabic speaking countries	160	42.9	29	Self-reported	Y-BOCS-SR, OCI-R	myMCT	6	Passive (waitlist)	No support, 0
**Moritz, Bernardini & Lion, 2019** [29]	Italian speaking population	80 (OCI-R); 11 (YBOCS)	63.4	41	Self-reported	Y-BOCS-SR, OCI-R	myMCT	6	Passive (waitlist)	Only answers if questions, 0
**Wootton et al., 2019** [61]	Australia	140	81.5	33.7	Y-BOCS scored at least 14, DOCS at least 7	Y-BOCS, DOCS	iCBT	8	Passive (waitlist)	Only answers if questions, 0
**Vogel et al., 2014** [62]	Norway	20	50	35.3	DSM-IV	Y-BOCS	Self-help book (PDF)^a^	12	Passive (Waitlist), Other treatments (video-conference assisted ERP)	Only answers if questions, 0
**Moritz & Russu, 2013** [56]	Russia	72	44.4	23.8	60 diagnosed by specialist, 12 self-diagnosed	Y-BOCS-SR, OCI-R	Association Splitting	4	Passive (Waitlist)	Only answers if questions, 0
**Schröder et al., 2020** [63]	Germany	128	76.6	40.22	Y-BOCS scored at least 8	Y-BOCS-SR	iCBT	8	Other treatments (face-to-face CBT)	No support, 0

*DSM-IV* Diagnostic and Statistical Manual of Mental Disorders Fourth Edition, *DSM-IV-TR* Diagnostic and Statistical Manual of Mental Disorders Fourth Edition - Text Revision, *Y-BOCS* Yale-Brown Obsessive-Compulsive Scale, *OCI-R* Obsessive-Compulsive Inventory-Revised, *DOCS* Dimensional Obsessive-Compulsive Scale, *t.-d.* technology-delivered, *iCBT* internet-administered CBT, *iPRT* internet-based progressive muscle relaxation, *BT Steps* computerized telephone system with 9 steps of CBT, *myMCT* Metacognitive training, *COMET* Competitive Memory Training, *TAU* treatment as usual; ^a^ = by Foa & Kozak (1997)

The duration of interventions ranged from 4 to 12 weeks. Six studies included follow-up assessments ranging from 12 to 24 weeks. OCD outcome measures were the Y-BOCS [27] in 17 studies, OCI-R [33] in ten studies and DOCS [34] in three studies. Pre-treatment mean scores for Y-BOCS ranged from 18.29 (Y-BOCS self-reported) to 25.80 (*M* = 25.96, *SD* = 7.61), for OCI-R from 19.68 (OCI-R self-reported) to 49.03 (*M* = 24.53, *SD* = 6.86) and for DOCS from 11.88 to 34.50 (*M* = 26.74, *SD* = 8.43). Primary OCD diagnosis was assessed by the Structured Clinical Interview (SCID) or the short version Mini-International Neuropsychiatric Interview (MINI) for DSM-IV and DSM-IV-TR (*k* = 7), self-report measures (*k* = 2), diagnosed prior by a mental health specialist (*k* = 5), self-report measures and diagnosed by a mental health specialist (*k* = 1) or a certain cut-off score of Y-BOCS and DOCS (*k* = 2). As mentioned above, two studies (i.e., [29, 30]) included a sample with a self-reported OCD diagnosis and two studies included a sample with a Y-BOCS score of at least 8 and with a DOCS subscale score of at least 7(i.e., [61, 63]). However, these studies were included as OCD measures indicated average scores above the cut-off-score (Y-BOCS: 8–23, indicating a mild to moderate OCD, see Goodman et al., 1989; DOCS: > 15, see [64]).

Technology-delivered interventions with CBT elements were Association Splitting (AS [65]) sent through email (*k* = 2), Metacognitive Training (myMCT [28]) with the help of a self-help book sent through email (*k* = 6), internet based CBT (iCBT, *k* = 7), a self-help book by Foa & Kozak (1997) delivered through email (*k* = 1), competitive memory training (COMET [66]) delivered through an internet based download (*k* = 1), a web-based CBT program (BTSteps [67]) with an IVR system and a self-paced workbook delivered through a telephone (*k* = 1). Control conditions included online non-directive supportive therapy (*k* = 1), face-to-face sessions (*k* = 2), relaxation therapy (*k* = 2), and treatment as usual (*k* = 1). Hauschildt, Schröder and Moritz (2016) provided the control conditions with psychoeducation which we considered as an active control condition [53]. Medication treatments were not coded.

### Study quality

Risks of bias were assessed according to the Cochrane Collaboration tool. A summary of the overall assessment of the within-trial risk of bias and the support for the judgement of selection bias and detection bias is provided in the supplementary material S1 Table. Three studies were at high risk of bias for the selection bias [56, 61, 62]. Risk of bias for the attrition bias was high in three studies as an ITT analysis was not conducted [28, 60, 63]. Additionally, there was a high risk of bias owing to incomplete outcome data reporting for five studies: missing means and standard deviations [56, 68], incomplete ITT results report [27, 30] and missing report of the amount of sample [29].

### Publication bias

**Fig. 2:**
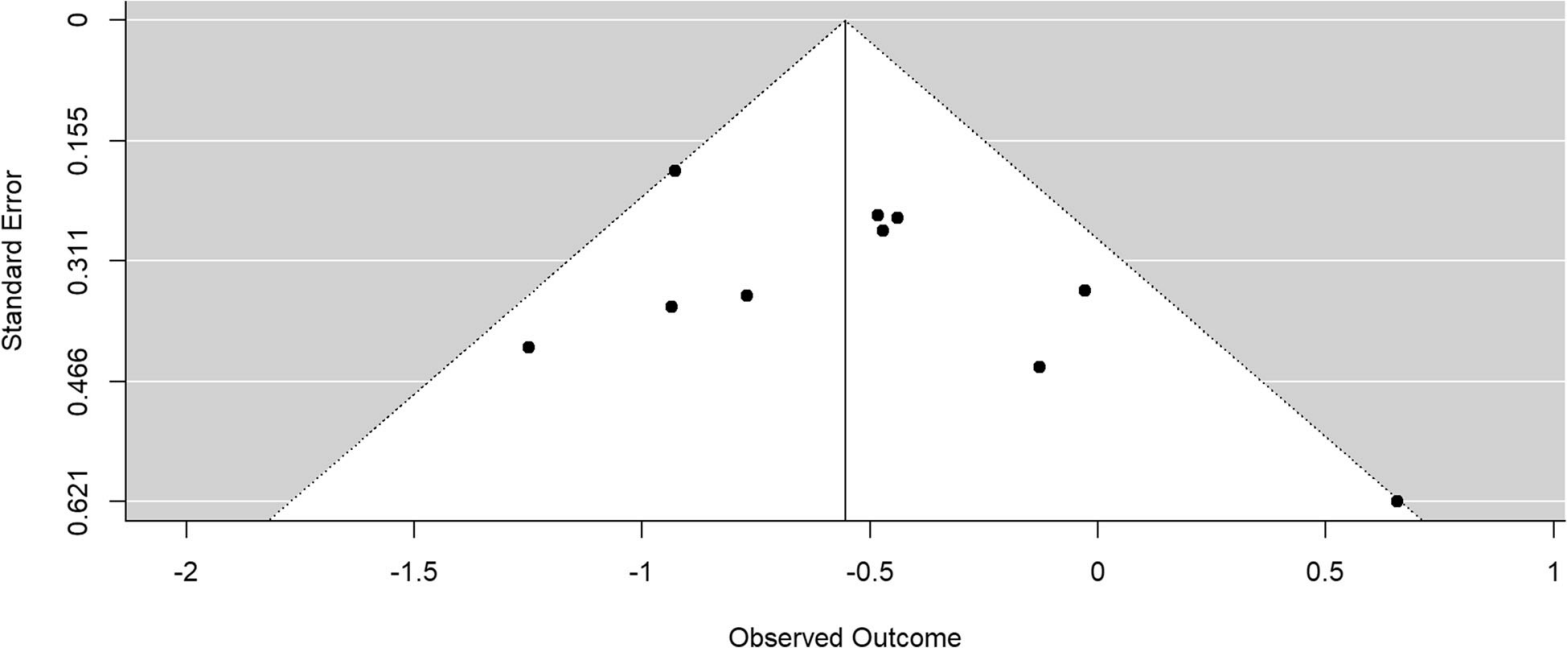
Funnel plot for remote CBT versus passive control groups with Y-BOCS and DOCS combined; test for funnel plot asymmetry: *t* = 1.3174, *df* = 8, *p* = 0.224

For determining the publication bias we examined the Funnel plot and conducted the Egger regression test. Regarding a comparison of outcomes for remote CBT versus passive control at post-treatment and comparing pre-post-treatment outcomes, the results of the Egger test were not statistically significant. The Funnel plot for remote CBT versus passive controls with Y-BOCS and DOCS combined is presented in Fig. 2. In terms of the comparison of remote CBT versus controls with other treatments only at post-treatment, the Egger test suggest a significant result for the funnel plot asymmetry (remote CBT versus controls with other treatments for Y-BOCS and DOCS combined: *t* = 2.7557, *df* = 6, *p* = 0.0330; remote CBT versus controls with other treatments for Y-BOCS: *t* = 3.0809, *df* = 5, *p* = 0.0274). However, the main results indicated no evidence of publication bias.

### Synthesis of results

Tables 2 and 3 present effect sizes (g) for each control group and the pooled effect size regarding the main effect of groups and the group-by-time interaction effect size. Results are presented for Y-BOCS only, Y-BOCS and DOCS combined and OCI-R only. As there were missing scores (means and SDs) for Moritz & Russu (2013) and Moritz et al. (2016) [56, 58], these studies did not provide enough information for inclusion in the quantitative analysis. Results of Moritz et al. (2016) showed a greater reduction in Y-BOCS score for the myMCT-groups (in the analysis combined) compared to the control group (complete cases for group-by-time interaction: *F* [1, 48] = 4.31, *p* = .044, *d* = 0.62 [62]). In their per protocol analysis, Moritz and Russu (2013) presented an improvement on the Y-BOCS score for group-by-time interaction (*F* [1, 45] = 4.50, *p* = 0.04, η^2^partial = 0.09 [53]).

**Table 2:** Group-by-time interaction effect

Study	Type of control group	Y-BOCS		Y-BOCS and DOCS combined		OCI-R	
		g	99% CI	g	99% CI	g	99% CI
**Andersson et al., 2012** [51]	Other treatments (online non-directive supportive therapy); *N* = 51	− 1.33	− 1.76 to − 0.89	− 1.33	− 1.76 to − 0.89		
**Greist et al., 2002 (a)** [52]	Other treatments (clinician guided face-to-face); *N* = 69	0.33	− 0.04 to 0.71	0.33	− 0.04 to 0.71		
**Greist et al., 2002 (b)** [52]	Other treatements (relaxation); *N* = 75	− 0.63	− 1.00 to − 0.26	− 0.63	− 1.00 to − 0.26		
**Hauschildt, Schröder& Moritz, 2016** [53]	Other treatments (only psychoeducation); *N* = 64	− 0.27	− 0.62 to 0.08	− 0.27	− 0.62 to 0.08		
**Kyrios et al., 2018** [55]	Other treatments (internet-based PMR); *N* = 90	− 0.54	− 0.88 to − 0.21	− 0.31	− 0.65 to 0.02		
**Mahoney et al., 2014** [60]	Other treatments (treatment as usual); *N* = 35			− 0.93	− 1.48 to − 0.38		
**Vogel et al., 2014 (b)** [62]	Other treatments (video-conference assisted ERP); *N* = 10	2.30	1.17 to 3.43	2.30	1.17 to 3.43		
**Schröder et al., 2020** [63]	Other treatments (face-to-face CBT); *N* = 64	− 0.25	− 0.65 to 0.15	− 0.25	− 0.65 to 0.15		
**Wootton et al., 2013** [57]	Passive (WLC); *N* = 17	−2.15	−3.09 to − 1.22	− 1.49	− 2.35to − 0.63		
**Herbst et al., 2014** [54]	Passive (WLC); *N* = 18	− 0.81	− 1.51 to − 0.11	− 0.81	− 1.51 to − 0.11	− 0.84	− 1.55 to − 0.14
**Schneider et al., 2014** [58]	Passive (WLC); *N* = 31	− 0.26	− 0.75to 0.23	− 0.26	− 0.75to 0.23	−0.19	− 0.68to 0.30
**Moritz et al., 2010** [28]	Passive (WLC); *N* = 43	− 0.24	−0.74to 0.25	− 0.24	−0.74to 0.25	− 0.29	−0.79to 0.20
**Moritz et al., 2018** [27]	Passive (WLC); *N* = 35	−0.70	− 1.24 to − 0.16	−0.70	− 1.24 to − 0.16	−0.28	− 0.81to 0.24
**Moritz & Jelinek, 2011** [59]	Passive (WLC); *N* = 23	− 0.91	− 1.63 to − 0.18	−0.91	−1.63 to − 0.18	−0.26	− 0.95 to 0.44
**Moritz et al., 2019** [29, 30]	Passive (WLC); *N* = 76	− 0.34	− 1.03 to 0.35	−0.34	−1.03 to 0.35	− 0.40	−0.92 to 0.13
**Moritz, Bernardini & Lion, 2019** [29]	Passive (WLC); *N* = 39	0.18	−1.01 to 1.37	0.18	−1.01 to 1.37	−0.04	−0.62 to 0.54
**Wootton et al., 2019** [61]	Passive (WLC); *N* = 75	−1.08	− 1.47 to − 0.70	−0.74	−1.12 to − 0.37		
**Vogel et al., 2014 (a)** [62]	Passive (WLC); *N* = 36	− 0.30	−1.18 to 0.59	−0.30	− 1.18 to 0.59		
**Overall estimate (remote treatment vs. passive controls)**		−0.66**	−1-08 to − 0.25	−0.57**	− 0.85 to − 0.13	−0.30*	− 0.56 to − 0.04
**Overall estimate (remote treatment vs. controls with other treatments)**		− 0.13	−1.12 to 0.87	−0.23	− 1.09 to 0.63		

Group-by-time interaction effect legend

*WLC* wait list control group, *Y-BOCS* Yale-Brown Obsessive-Compulsive Scale, *OCI-R*, Obsessive-Compulsive Inventory-Revised, *DOCS* Dimensional Obsessive-Compulsive Scale, *CI* Confidence interval. Significance codes = *** < 0.001; ** < 0.01; * < 0.05

**Table 3:** Main effect of groups

Study	Type of control group	Y-BOCS		Y-BOCS and DOCS combined		OCI-R	
		g	99% CI	g	99% CI	g	99% CI
Andersson et al., 2012 [51]	Other treatments (online non-directive supportive therapy); *N* = 51	−1.11	− 1.53 to − 0.69	−1.11	− 1.53 to − 0.69		
Greist et al., 2002 (a) [52]	Other treatments (clinician guided face-to-face); *N* = 69	0.21	− 0.17 to 0.58	0.21	−0.17 to 0.58		
Greist et al., 2002 (b) [52]	Other treatments (relaxation); *N* = 75	−0.73	−1.10 to − 0.36	−0.73	−1.10 to − 0.36		
Hauschildt, Schröder& Moritz, 2016 [53]	Other treatments (only psychoeducation); *N* = 64	− 0.10	−0.45 to 0.25	− 0.10	−0.45 to 0.25		
Kyrios et al., 2018 [55]	Other treatments (internet-based PMR); *N* = 90	−0.55	−0.89to − 0.21	−0.55	− 0.89, − 0.21		
Mahoney et al., 2014 [60]	Other treatments (treatment as usual); *N* = 35			− 0.74	−1.27 to − 0.20		
Vogel et al., 2014 (b) [62]	Other treatments (video-conference assisted ERP); *N* = 10	2.06	0.97 to 3.14	2.06	0.97 to 3.14		
Schröder et al., 2020 [63]	Other treatments (face-to-face CBT); *N* = 64	−0.23	−0.64 to 0.17	− 0.23	−0.64 to 0.17		
Wootton et al., 2013 [57]	Passive (WLC); N = 17	−1.79	− 2.67to − 0.90	− 1.25	−2.08to − 0.42		
Herbst et al., 2014 [54]	Passive (WLC); N = 18	−0.77	−1.47 to − 0.07	−0.77	−1.47 to − 0.07	−0.84	−1.54 to − 0.13
Schneider et al., 2014 [58]	Passive (WLC); N = 31	−0.48	− 0.98to 0.01	−0.48	− 0.98to 0.01	−0.23	− 0.72to 0.26
Moritz et al., 2010 [28]	Passive (WLC); N = 43	− 0.44	−0.94to 0.06	− 0.44	−0.94to 0.06	− 0.46	−0.96to 0.04
Moritz et al., 2018 [27]	Passive (WLC); N = 35	−0.47	−1.00 to 0.06	− 0.47	− 1.00 to 0.06	−0.31	− 0.84 to 0.22
Moritz & Jelinek, 2011 [59]	Passive (WLC); N = 23	− 0.93	− 1.66 to − 0.21	−0.93	− 1.66 to − 0.21	−0.41	−1.11 to 0.29
Moritz et al., 2019 [29, 30]	Passive (WLC); N = 76	−0.03	− 0.71 to 0.66	−0.03	− 0.71 to 0.66	−0.40	− 0.93 to 0.12
Moritz, Bernardini & Lion, 2019 [29]	Passive (WLC); N = 39	0.66	−0.56 to 1.87	0.66	−0.56 to 1.87	− 0.02	−0.60 to 0.55
Wootton et al., 2019 [61]	Passive (WLC); N = 75	−1.01	− 1.40 to − 0.63	−0.93	−1.66 to − 0.21		
Vogel et al., 2014 (a) [62]	Passive (WLC); N = 36	− 0.13	− 1.01 to 0.75	−0.13	− 1.01 to 0.75		
Overall estimate (remote treatment vs. passive controls)		−0.59*	−0.99 to − 0.18	−0.55**	− 0.87 to − 0.24	−0.36*	− 0.62 to − 0.09
Overall estimate (remote treatment vs. controls with other treatments)		− 0.14	−1.02 to 0.74	−0.22	− 0.97 to 0.53		

Main effect of groups legend

*WLC* wait list control group, *Y-BOCS* Yale-Brown Obsessive-Compulsive Scale, *OCI-R* Obsessive-Compulsive Inventory-Revised, *DOCS* Dimensional Obsessive-Compulsive Scale, *CI* Confidence interval. Significance codes = *** < 0.001; ** < 0.01; * < 0.05

#### Group-by-time interaction effect

**Fig. 3:**
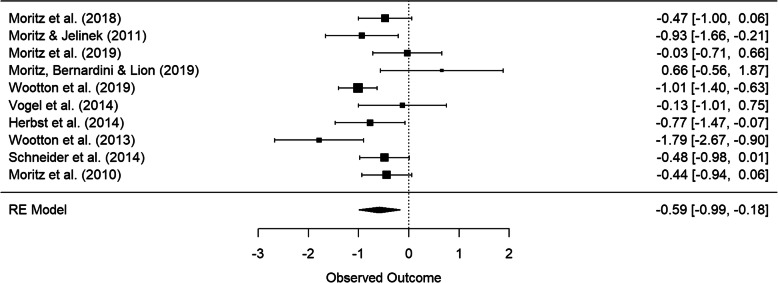
Forest plot for remote CBT versus passive control groups reporting Y-BOCS outcomes for the change from pre- to post-treatment

Table 2 outlines the group-by-time interaction effect sizes for the studies included in the quantitative synthesis. Group-by-time interaction indicates whether low intensity technology-delivered CBT results in significant improvement in OCD symptoms from pre- to post-treatment compared to the corresponding pre-post changes in the control group. Outcomes were analyzed separately for passive controls and active controls. Regarding remote treatment versus passive controls for Y-BOCS only, a total of 10 comparisons were drawn. The pooled effect size indicated a significant moderate to large effect of − 0.66 (95% CI = [− 1.08, − 0.25]; *p* = 0.006, *Q* = 23.76, *df* = 9, *p* = 0.005, *I*^2^ = 68.62%; Fig. 3). Referring to the results of remote treatment versus passive controls for Y-BOCS and DOCS combined, there was a significant pooled effect size of − 0.57 (95% CI = [− 0.85, − 0.30], *p* = 0.001, *Q* = 12.28, *df* = 9, *p* = 0.20, *I*^2^ = 30.74%). For OCI-R only there was a small significant effect size (*g* = − 0.30, 95% CI = [− 0.56, − 0.04], *p* = 0.03, *Q* = 3.41, *df* = 6, *p* = 0.76, *I*^2^ = 0%).

The overall effect size of the comparisons between remote treatment versus controls with other treatments was not statistically significant and there was an evidence for the presence of heterogeneity for Y-BOCS only (*g* = − 0.13, 95% CI = [− 1.12, 0.87], *p* = 0.77, *Q* = 57.24, *df* = 6, *p* < 0.0001, *I*^2^ = 96.23%) and Y-BOCS and DOCS combined (*g* = − 0.23, 95% CI = [− 1.09, 0.63], *p* = 0.55, *Q* = 61.01, *df* = 7, *p* < 0.0001, *I*^2^ = 95.63%). There were no studies measuring OCI-R.

#### Main effect of group

**Fig. 4:**
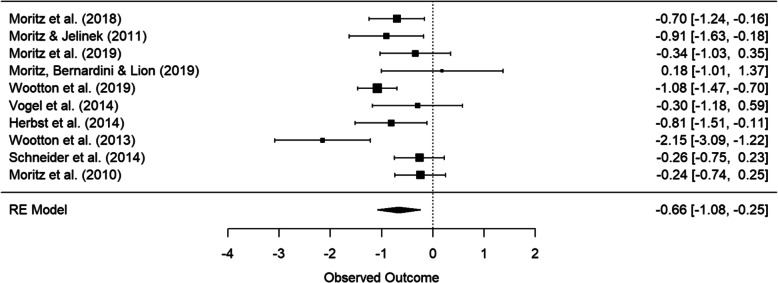
Forest plot for remote CBT versus passive control groups at post-treatment reporting Y-BOCS outcomes

Between-group effect sizes at post-treatment for the included studies in the quantitative synthesis are presented in Table 3. Passive controls and active controls were analyzed separately. According to passive controls, a total of 10 comparisons were drawn for Y-BOCS only and for Y-BOCS and DOCS combined and a total of 7 comparisons for OCI-R. Heterogeneity was present for Y-BOCS only (*Q* = 20.98, *df* = 9, *p* = 0.01, *I*^2^ = 67.56%). For Y-BOCS and DOCS combined (*Q* = 14.97, *df* = 9, *p* = 0.09, *I*^2^ = 47.39%) as well as for OCI-R only (*Q* = 3.57, *df* = 6, *p* = 0.74, *I*^2^ = 0%), there was statistically no evidence for heterogeneity. The significant pooled effect sizes were moderate to large for Y-BOCS only effect (*g* = − 0.59, 95% CI = [− 0.99, − 0.18], *p* = 0.01; Fig. 4) and Y-BOCS and DOCS combined (*g* = − 0.55, 95% CI = [− 0.87, − 0.24], *p* = 0.003) and small for OCI-R (*g* = − 0.36, 95% CI = [− 0.62, − 0.09], *p* = 0.02).

According to controls with other treatments, a total of 7 comparisons were drawn for Y-BOCS only and a total of 8 comparisons for Y-BOCS and DOCS combined with a presence of heterogeneity (Y-BOCS: *Q* = 47.69, *df* = 6, *p* < 0.0001, *I*^2^ = 95.22%; Y-BOCS and DOCS combined: *Q* = 49.55, *df* = 7, *p* < 0.0001, *I*^2^ = 94.32%). The pooled effect sizes were not significant (Y-BOCS: *g* = − 0.14, 95% CI = [− 1.02, 0.74], *p* = 0.71; Y-BOCS and DOCS combined: *g* = − 0.22, 95% CI = [− 0.97, 0.53], *p* = 0.51). There were no studies measuring OCI-R.

#### Moderator analyses and sub analyses

The following results are only presented according to YBOCS outcomes and passive controls (*n* = 10). Sample age did not significantly moderate the overall effect of remote CBT with low personal contact intensity on OCD symptoms (*b* = − 0.03, *SE* = 0.06, 95% CI = [− 0.18, 0.12], *p* = 0.69), whereas women percentage showed a small significant moderating effect (*b* = − 0.03,*SE* = 0.01, 95% CI = [− 0.06, − 0.002], *p* = 0.04). Results demonstrated no significant moderating effect bythe duration of intervention (*b* = − 0.06, *SE* = 0.08, *p* = 0.46,95% CI = [− 0.25, 0.12]). Irrespective of the support conditions (yes versus no), we could not find evidence of a difference in symptom outcomes (*b* = − 0.84, *SE* = 0.41, 95% CI = [− 1.79, 0.11], *p* = 0.08). Moreover, we could not find evidence of a difference in symptom outcomes if samples were diagnosed according to DSM or not (*b* = − 0.51, *SE* = 0.41, 95% CI = [− 1.45, 0.43], *p* = 0.25). Study quality of included studies was not a significant moderator of the overall effect (*b* = 0.14, *SE* = 0.16, 95% CI = [− 0.22, 0.50], *p* = 0.41). Conducting a sub analysis including only studies with low risk of bias, results showed larger significant improvement in OCD symptoms from pre- to post-treatment compared to the corresponding pre-post changes in the control group (YBOCS: *g* = − 0.88, 95% CI = [− 1.56, − 0.21], *p* = 0.02, *Q* = 16.01, *df* = 5, *p* = 0.007, *I*^2^ = 74.66%; YBOCS and DOCS combined: *g* = − 0.70, 95% CI = [− 1.12, − 0.28], *p* = 0.008, *Q* = 7.59,*df* = 5, *p* = 0.18, *I*^2^ = 37.90%). However, heterogeneity was present for the analysis with YBOCS only. For between-group effect sizes at post-treatment, results showed as well larger significant effect sizes (YBOCS: *g* = − 0.84, 95% CI = [− 1.36, − 0.31], *p* = 0.009, *Q* = 9.83, *df* = 5, *p* = 0.08, *I*^2^ = 58.01%; YBOCS and DOCS combined: *g* = − 0.77, 95% CI = [− 1.11, − 0.43], *p* = 0.002, *Q* = 5.51,*df* = 5, *p* = 0.36, *I*^2^ = 9.44%).

The forest plot of the main effect of time (change) of Y-BOCS for only the passive control groups is shown in the supplementary material S1 Figure.

## Discussion

The purpose of this study was to provide an extension of the available meta-analyses of technology-delivered CBT for OCD. Specifically, we aimed to evaluate the efficacy of only low personal contact intensity treatments. We found moderate effect sizes for group-by-time interaction and main effect of group when comparing remote CBT with low personal contact intensity versus passive controls. Results indicate that delivering technology-delivered CBT with low personal contact can be efficacious in reducing OCD symptoms. The sub-analysis on OCI-R demonstrated only a small significant overall effect. However, this could be due to the small amount of studies (*N* = 7) or due to a smaller sensitivity to change compared to Y-BOCS [69].

When comparing remote CBT and controls with other treatments, neither the group-by-time interaction nor the main effect of group pointed to significant differences between conditions. In this context, it is important to note that we could only retrieve seven studies including control groups with other treatments and secondly heterogeneous controls with other treatments were utilized in these studies (i.e., face-to-face treatments, internet-based relaxation programs or technology-delivered treatments with high personal contact intensity).

### Comparison with the literature

Our findings are in line with results of other systematic reviews and meta-analyses supporting the conclusion that technology-delivered CBT is a promising efficacious treatment in comparison to passive control conditions [70]. General meta-analytic reviews of internet interventions indicate that guided technology-delivered interventions are as effective as face-to-face interventions [71]. Due to low numbers of studies with control conditions with other treatments and large heterogeneity in designs, the present meta-analysis could not compare face-to-face CBT with technology-delivered CBT with low contact intensity.

Meta-analyses on internet-delivered CBT for OCD reported that guided internet-based interventions lead to a greater symptom reduction [21] and a smaller drop-out rate than unguided versions of treatments [20]. In their meta-analysis on internet-based psychological interventions for depression, Johannson and Andersson (2012) shed light on the influence of time of therapist-guidance on symptom outcome [72]. They suggest a linear positive effect, that is, no therapist contact showed a between-group Cohen’s d effect size of *d* = 0.21, whereas therapist contact before treatment resulted in *d* = 0.44 and during treatment in *d* = 0.58. Therapist contact before and during treatment produced an effect size of *d* = 0.76.

Somewhat inconsistent with these findings, our results emphasize that not only highly frequented and intensive clinician-guidance can lead to a symptom reduction but also low personal contact intensity during treatment leads to moderate to large effect size of approximately 0.6. Even though recent research indicates that therapist guidance is related to better outcomes in terms of symptom reductions, we found no evidence for a moderating effect of support. In light of the included interventions with only low personal contact intensity support or no contact at all, this may reflect that pure self-help interventions are as efficacious as interventions with low personal contact intensity. This would imply that an asynchronous contact, namely answering time-delayed, automatically generated answers and a low frequency can be as well sufficient for ensuring successful application. However, obviously more rigorous studies are needed to shed more light on the optimal frequency, type and dose of therapeutic contact in technology-delivered psychotherapy for OCD.

Results suggest that technology-delivered interventions for OCD without any therapist contact might be efficacious, too. Therapist drift is a common pattern during face-to-face CBT sessions [73], hence, reduced therapist contact during interventions coulddecrease this phenomenon potentially. Moreover, technology-delivered interventions increase cost-effectiveness [74].

### Strengths and limitation

As there were many recent randomized controlled trials, providing an updated meta-analysis was highly recommended [75]. Therefore, one strength of the present article is the inclusion of seven recently published RCTs [27, 29, 53, 55, 61, 63, 68]. The present article is one of the first articles in this line of research to include only RCTs as well as a sample comprising only adults. Considering generalizability, it is important to note that trials were conducted in different countries and continents from various independent research groups. Additionally, we included only RCTs investigating the efficacy of evidence-based cognitive behavioral interventions leading to a rather homogenous group of high-quality interventions. Another strength is that we touch upon an economic issue. Technology-delivered interventions with low or no clinician-contact present a cost-effective alternative to mainstream face–to-face interventions, particularly when efficacy is indeed shown equivalent as more empirical evidence emerges. Particularly for resource-scarce contexts (e.g., limited number of mental health specialists available, long waiting periods etc.) technology-based interventions present the probably most feasible, low-threshold, scalable option at hand.

However, our paper has several limitations. A major limitation of the present review concerns the small number of integrated trials as well as small samples sizes within several trials. Particularly, results from the moderator analyses should be interpreted with caution. Further investigations with greater sample size are required. Moreover, controls with other treatments varied widely. This leads to conclusion that the meaningfulness of the interpretability should considered with caution. Waiting list conditions are generating higher effect sizes for CBT than other conditions such as no treatment [76]. This may becaused by differences in expectations, as waiting list control group are waiting for a desired treatment may lead to negative effects. Consequently, our analyses might overestimate the effects of technology-delivered CBT since many trials used waiting list control groups.

One of the key problems in technology-based intervention research is the lack of an appropriate psychological diagnostic process with reference to well-known classification systems [77]. A limitation of this meta-analysis is that studies were also included when diagnoses were based on self-report measures. These trials may have included subclinical populations. However, our moderator analysis on types of diagnostic assessments indicated no evidence for a moderating effect.

An inherent limitation in this field of research concerns missing follow-up data. Only five studies included a follow-up measure precluding a meta-analytic review. As such, no conclusions could be drawn for the long-term efficacy of technology-delivered CBT for OCD. We urge researchers and funding bodies for the inclusion of long-term follow-up measures to address this limitation. Another point is that there was no controlling for concurrent medication during treatments. Hence, efficacy could be influenced, among other factors, by psychotropic drugs.

The current quantitative synthesis identified studies with low personal contact intensity, hence, we focused on minutes spent per intervention and participant. Nonetheless, a limitation could be that interventions had a varying length and minutes per week were not considered. Thus, the definition of 100 min per intervention and per participant could cause a false effect in consideration of the length of intervention. A further limitation of this meta-analysis is that we failed to preregister the protocol. That is, we cannot prove that we maintained our a priori defined methods and hypotheses. Moreover, a limitation of the present study is that the study selection was performed by a single author. That is, we may have missed relevant literature. However, we tried to make sure that we included all relevant literature by also checking recent related meta-analyses.

## Conclusions

Technology-delivered CBT for OCD with low personal contact intensity could save financial costs, clinician time, and could provide evidence-based CBT to individuals suffering from obsessions and compulsions who are not willing to start a face-to-face therapy or who do not have the possibility because of financial or long-distance problems. Furthermore, this specific type intervention is not stigma associated but anonymous and provides autonomy to take steps in an own pace and at a chosen place. As such, it could be provided as a stepped care model which involves the application of a less intensive step, i.e. technology-delivered CBT with low personal contact intensity or no clinician contact, before starting an intensive face-to-face treatment. In summary, as a consequence of lower therapist time that is involved, technology-delivered CBT with low personal contact intensity has the potential benefit of cost and time savings for healthcare providers. Additionally, patients with avoidance behavior due to shame, stigma or due to the need for more autonomy may profit from these interventions as well. It is important that we focus on evidence-based high standardized interventions for overcoming barriers and for providing interventions for every human condition. Further investigations through use of RCTs with technology-delivered interventions providing low personal contact intensity guidance, particularly investigating comparison to active and passive control groups, is required.

## Supplementary Information

**Additional file 1:** **Table S1.** Assessed risks of bias = selection bias, attrition bias, detection bias and reporting bias. Scores for overall assessment = low (1), unclear (2) and high (3). **Fig. S1.** Forest plot of the main effect of time (change) of Y-BOCS for only the passive control groups.

## Data Availability

All the materials including screening sheet, data extractions sheet, study quality assessments and analyses are available from our Open Science Framework (OSF) data repository (https://osf.io/7upt4/?view_only=34f63ec15df545f0a8abbc6bdf14d77d).

## Abbreviations

OCD: Obsessive-compulsive disorder
CBT: Cognitive behavioral therapy
RCT: Randomized controlled trial
ERP: Exposure and response prevention
PICOS: Population, Intervention, Comparison, Outcome, and Study design
PRISMA: Preferred Reporting Items for Systematic Reviews and Meta-analysis
NICE: National Institute for Health and Care Excellence
CR: Cognitive restructuring
IVR: Interactive voice response
Y-BOCS: Yale-Brown Obsessive-Compulsive Scale
OCI-R: Obsessive-Compulsive-Inventory-Revised
DOCS: Dimensional Obsessive-Compulsive Scale
OSF: Open Science Framework
vCBT: Videoconferencing-administered CBT
tCBT: Telephone-administered CBT
cCBT: Computerized CBT
iCBT: Internet-administered CBT
bCBT: Bibliotherapy administered CBT
HCI: High contact intensity
RCI: Reduced contact intensity
ASH: Self-help with assistance to ensure engagement with the tool
PSH: Pure self-help
ITT: Intention to treat
