# Supplementary material for: Low intensity technology-delivered cognitive behavioral therapy for obsessive-compulsive disorder: a meta-analysis
Source: BMC Psychiatry. 2021 Jun 30;21:322. doi: 10.1186/s12888-021-03272-5 (PMC8243493; doi:10.1186/s12888-021-03272-5)
Supplement: Supplementary file 1 — Additional file 1: Table S1. Assessed risks of bias = selection bias, attrition bias, detection bias and reporting bias. Scores for overall assessment = low (1), unclear (2) and high (3). Fig. S1. Forest plot of the main effect of time (change) of Y-BOCS for only the passive control groups. [file 12888_2021_3272_MOESM1_ESM.docx]

| **Supplementary material**  S1 Table. Cochrane assessment of risk of bias for the 18 studies. | | | |
| --- | --- | --- | --- |
| Study | **Overall risk of bias assessment** | **Risk of bias and support for judgement of selection bias** | **Risk of bias and support for judgement of detection bias** |
| Andersson et al., 2012 | 0 | Low risk of bias. Participants were randomized (www.random.org) with a 1:1 ratio by an independent person who was not involved in the study. | Low risk of bias. The assessors were blinded to treatment allocation at the post-treatment interview. Blinding integrity  was tested. |
| Greist et al., 2002 | 1 | Unclear risk of bias. Participants were randomized but the authors did not report the method used to generate and conceal the allocation sequence. | Low risk of bias. Also 90 of the 218 participants were rated on the clinician-administered YBOCS by a rater blind to treatment, there was no significant difference between self-rating and clinician administered rating. |
| Hauschildt, Schröder& Moritz, 2016 | 1 | Unclear risk of bias. Participants were randomized but the authors did not report the method used to generate and conceal the allocation sequence. | Low risk of bias. Assessors were blind to treatment condition. |
| Herbst et al., 2014 | 0 | Low risk of bias. Participants were randomized to one of the two experimental conditions using a block randomization with randomly permuted blocks. | Low risk of bias. Self-reported measure. |
| Kyrios et al., 2018 | 0 | Low risk of bias. Participants were randomized post baseline interview into the active treatment (iCBT) or control condition (iPRT) using an independent automated computer-generated sequence that could not be forecast or modified by the researchers. Coordinated by an independent statistician. | Low risk of bias. Posttreatment assessors were blind to treatment condition |
| Moritz et al., 2016 | 4 | Unclear risk of bias. Randomization was performed after baseline assessment; individuals were allocated to treatment arms in a computer-generated random order according to date of participation. | Low risk of bias. Self-reported measure. |
| Wootton et al., 2013 | 1 | Low risk of bias. Participants were randomized; randomisation sequence was computer-generated by an independent overseas colleague using www.random.org. | Unclear risk of bias. No further information. |
| Schneider et al., 2014 | 0 | Low risk of bias. Randomization through UniPark. | Low risk of bias. Self-reported measure. |
| Moritz et al., 2010 | 3 | Unclear risk of bias. Participants who left e-mail addresses were allocated to a random plan but the authors did not report the method used to generate and conceal the allocation sequence. | Low risk of bias. Self-reported measure. |
| Moritz et al., 2018 | 3 | Unclear risk of bias. Participants were randomized to one of the two conditions based on the date of participation butthe authors did not report the method used to generate and conceal the allocation sequence. | Low risk of bias. Self-reported measure. |
| Moritz & Jelinek, 2011 | 1 | Unclear risk of bias. Participants completing the survey were allocated to the experimental or control groups according to a random plan (no stratification) butthe authors did not report the method used to generate and conceal the allocation sequence. | Low risk of bias. Self-reported measure. |
| Mahoney et al., 2014 | 2 | Low risk of bias. Randomisation was based on a random number sequence generated at www.random.org; Concealment of allocation was maintained until the applicant met all inclusion criteria and an offer of participant was made. | Low risk of bias. Self-reported measure. |
| Moritz et al., 2019 | 3 | Unclear risk of bias. Participants were randomized to one of the two conditions (allocation was according to the date of participation as displayed in the so-called trigger e-mail). But there is no further information about the exact randomization plan. | Low risk of bias. Self-reported measure. |
| Moritz, Bernardini& Lion, 2019 | 3 | Unclear risk of bias. Participants were randomized to the conditions according to the date of participation following a randomization plan. The allocation procedure is best described as centralized assignment. But there is no further information about the exact randomization plan. | Low risk of bias. Self-reported measure. |
| Wootton et al., 2019 | 2 | High risk of bias. Randomization on a 1:1 basis to either the immediate or delayed treatment using an online random number generator (www.random.org) but there was no allocation concealment. | Low risk of bias. Self-reported measure. |
| Vogel et al., 2014 | 2 | High risk of bias. Participants were randomized butthe authors did not report the method used to generate and conceal the allocation sequence. There was a significant difference between groups. | Low risk of bias. Participants were interviewed per telephone by a psychologist blinded as to treatment condition. |
| Moritz &Russu, 2013 | 4 | High risk of bias. Participants were randomly allocated to either the experimental or control group with no further stratification applied. | Low risk of bias. Self-reported measure. |
| Schröder et al., 2020 | 2 |  |  |
| Cochrane assessment of risk of bias for the 18 studies legend.Assessed risks of bias = selection bias, attrition bias, detection bias and reporting bias. Scores for overall assessment = low (1), unclear (2) and high (3). | | | |


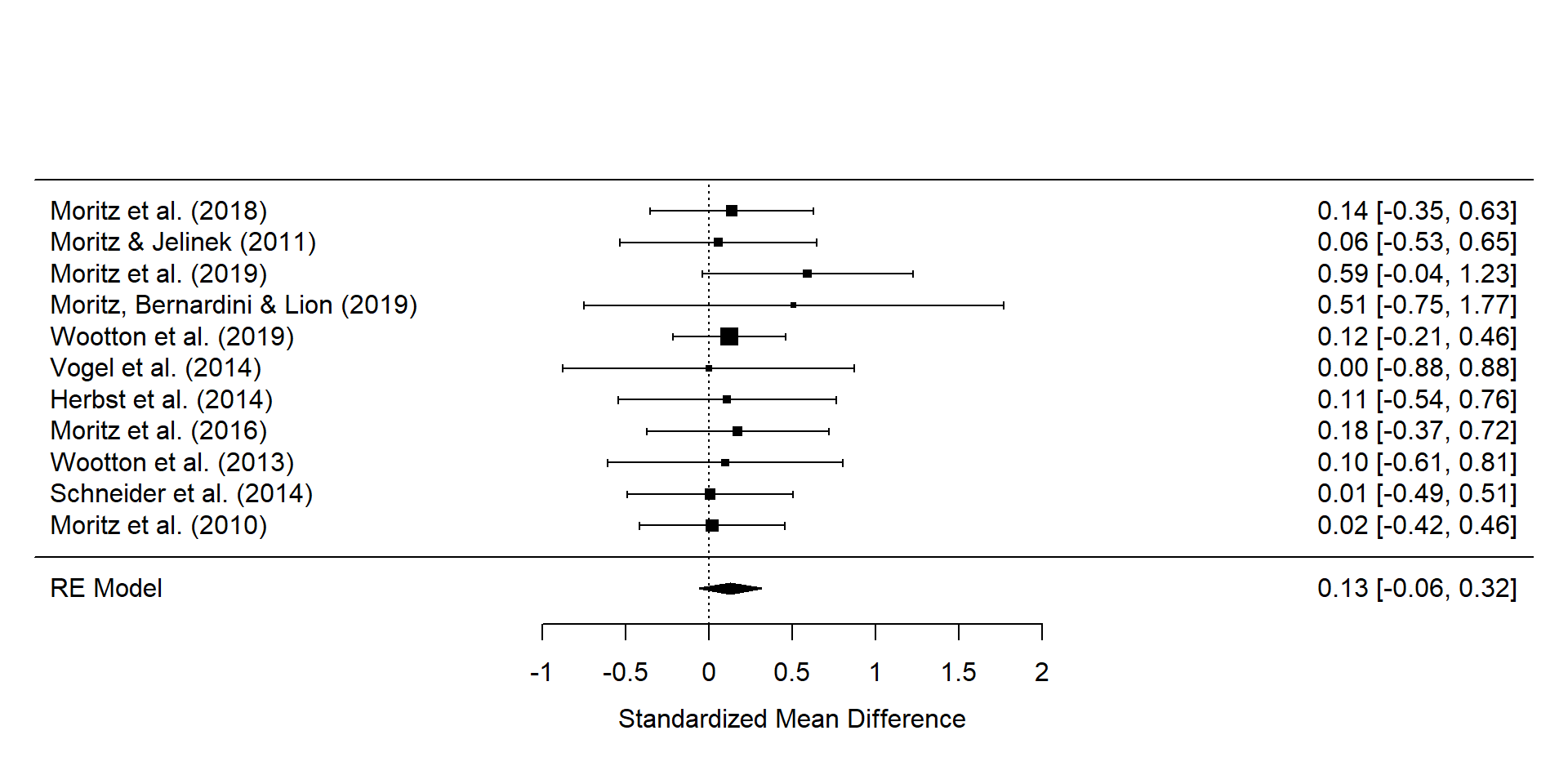


**S1 Fig.Forest plot.**Forest plot of the main effect of time (change) of Y-BOCS for only the passive control groups.
